# Child rearing or childbearing? Risk of cardiovascular diseases associated to parity and number of children

**DOI:** 10.1186/s12889-023-17119-z

**Published:** 2024-01-23

**Authors:** Angelo d’Errico, Dario Fontana, Carlotta Sacerdote, Chiara Ardito

**Affiliations:** 1 Epidemiology Unit Piedmont Region ASL TO3, Grugliasco (TO), Italy; 2 Unit of Cancer Epidemiology, University of Turin, Turin, Italy; 3 Centre for Cancer Epidemiology and Prevention (CPO Piemonte), Turin, Italy; 4 Competence Centre On Microeconomic Evaluation (CC-ME), European Commission, Joint Research Centre (JRC), Ispra, Italy

**Keywords:** Cardiovascular diseases, Children, Childrearing, Childbearing, Socioeconomic disadvantage, Double burden

## Abstract

**Background:**

An increased risk of cardiovascular diseases (CVD) has been associated with women’s parity, but whether or not this association reflects a direct pregnancy effect, or exposure to factors related to childrearing, still appears unclear. We assessed the CVD risk associated with number of children separately by gender and tested effect modification by socioeconomic position (SEP) and employment status, in order to elucidate the possible mechanisms underlying this association.

**Methods:**

The study population was composed of 20,904 men and 25,246 women who were interviewed in one of two National Health Surveys conducted in 2000 and 2005 in Italy. These subjects were followed for CVD incidence up to 2014 through record-linkage with national archives of mortality and hospitalisations. CVD risk was estimated by Cox regression models that were adjusted for socio-demographics, perceived health, lifestyles, biological CVD risk factors and for other potential confounders.

**Results:**

CVD incidence was significantly increased among men with 3 or more children (HR = 1.26, 95% CI: 1.02–1.56) and among women with 2 and with 3 or more children (HR = 1.42, 95% CI: 1.10–1.83; and HR = 1.39, 95% CI: 1.03–1.87, respectively) compared to subjects without children and no significant gender differences were observed. Subjects with lower SEP displayed stronger associations with parity and a higher number of children for both genders; by contrast, no modifying effect of employment status was observed.

**Conclusions:**

Taken together, the significant association between higher parity and CVD risk in both genders, and the higher risk of CVD associated with higher parity among lower SEP parents, suggests that childrearing has a potential effect on the development of CVD that is more pronounced among disadvantaged families, although a concurrent effect of childbearing cannot be completely excluded.

**Supplementary Information:**

The online version contains supplementary material available at 10.1186/s12889-023-17119-z.

## Introduction

The relationship between parity and risk of cardiovascular diseases (CVD) in women still appears to be controversial in the literature. Reviews of studies conducted previously concluded that there was no difference in risk between parous and nulliparous women [1, 2], but the results of more recent studies seem to indicate that parous women do carry a higher risk. The most recent review on the subject estimated an increase in CVD risk by 14% for ever parity, as well as an increase by 4% for each additional child among parous women, although with meta-ORs being affected by substantial heterogeneity [3]. In this study, Li et al. [3] found a non-linear relationship between number of children and CVD risk, with slightly increased risks for one or two children and a steeper, linear relationship for three children and above.

Pregnancy is a condition in which many biological changes, mostly temporary, occur in a woman’s body, on the metabolic and the cardiovascular system in particular, which may have a future impact on women’s cardiovascular health, such as weight gain, left ventricular hypertrophy, increased lipid levels and insulin resistance [4]. Moreover, adverse pregnancy outcomes, including gestational hypertension and preeclampsia, gestational diabetes, placental abruption, prematurity, and low birth weight, have been found to increase the risk of future CVD among women, although the mechanisms underlying this effect appear still unclear [5–7]. A review on the risk of CVD associated with common pregnancy complications (low birth weight, preterm delivery, gestational diabetes and preeclampsia), concluded that they are important determinants of future CVD, each approximately doubling their incidence, and that they would explain most of the association with parity [8].

Several risk factors for CVD, independent from adverse pregnancy outcomes, have also been found to be positively associated with parity, including hypertension [9], diabetes [10], high BMI [11], metabolic syndrome, high triglycerides and low HDL cholesterol levels [12]. These CVD risk factors have, thus, been proposed as possible mediators of parity on CVD risk, even though very few studies have investigated this issue [13].

However, it has been suggested that social factors related to childrearing, such as increased exposure to physical and mental stressors associated with childcare and responsibility for domestic chores or selection into parity (rather than only biological factors involved in childbearing), may also have an impact on women’s future health. The comparison of the associations between health and number of births among both women and men separately [14, 15] or among biological parents and adoptive parents [16] have been proposed as possible ways through which to disentangle the two potential mechanisms. Several epidemiological studies have examined the CVD risk associated with a person’s number of children in both women and men, on the basis that, because childbearing can have an impact on CVD among women exclusively, the presence of an association in both sexes would demonstrate that social factors are also involved. Most of these studies found similar associations in men and women, thereby supporting the childrearing hypothesis [11, 17–21], although no association was found among men in a few studies [22–24].

Furthermore, some studies found that the association with having children was limited to women who were in employment [25, 26], thereby suggesting that the increased CVD risk seen among women with higher parity would be due to the double burden imposed by the combination of both paid work and family responsibilities, which would overload them both physically and mentally [27, 28].

In addition, sons and daughters may have a different impact on women’s health, as found by an Italian study in which the association between presence of children in the household and risk of coronary heart disease differed according to sex of the offspring, with sons carrying a higher risk [26].

To fill some of these gaps in the current literature and to contribute to the elucidation of the relationship between parity and CVD and the potential underlying mechanisms, we conducted a study of the risk of hospitalisation or death from CVD associated with parity in a large representative sample of Italian women and men. Our main objective was to elucidate the role of social and cultural mechanisms as potential drivers of the association between CVD and having children. In particular we aimed to:

1. examine the association between CVD incidence and parity by sex and to evaluate whether or not the association with parity differs between women and men, given that this would indicate an effect of childbearing, rather than childrearing.
2. assess among both men and women if the association with parity is modified by socioeconomic position (SEP), which instead would indicate an effect of childrearing rather than childbearing.
3. assess if the association with parity is modified by employment status among women and to test the “double burden” hypothesis, which would also indicate an effect of childrearing rather than childbearing.

This latter analysis was limited to housewives and employed women, due to the low number of subjects who were unemployed or with other professional condition in both genders, and of housekeepers among men.

## Materials and methods

### Data collection

The study population consisted of women and men aged 25–45 years, married and living with their partner, who participated in either the 1999–2000 or the 2004–2005 editions of the Italian National Health Interview Survey (NHIS); this survey is carried out approximately every five years by the Italian National Institute of Statistics (Istat).

Both incarnations of the NHIS were conducted on representative samples of the resident population in Italy (52,332 families and 140,011 individuals in the 1999–2000 edition and 50,474 families and 128,040 individuals in the 2004–2005 edition). Data were collected by means of paper-and-pencil interviewing (PAPI) and were carried out on a quarterly basis in order to eliminate the seasonal effect on health. In the 1999–2000 NHIS, participation was 87% and in the 2004–2005 NHIS it was 83%. The survey provides detailed information on both individual and household socioeconomic status and employment, lifestyles (e.g., smoking, physical activity, overweight and obesity) as well as on health conditions, including long-term chronic diseases, disability, self-perceived health and on the use of health services.

A follow-up on the two cohorts was conducted from the date of the interview until the end of 2014 by means of record linkage with the Istat National Registry of Mortality and with the Italian Ministry of Health’s National Hospital Discharge Database, respectively [29]. The Istat National Registry of Mortality registers every death that occurred, in addition to the causes thereof. The Ministry of Health’s National Hospital Discharge Database contains information concerning all hospital admissions, including type of access to hospitalisation, diagnostic codes and any surgery and diagnostic/therapeutic procedures. The follow-up of the two pooled cohorts, called the Italian Longitudinal Study, was authorised within the National Statistical Programme (PSN code: IST-02566) with the aim of prospectively following up the samples of the NHIS for both mortality and morbidity. All data were fully anonymised prior to our accessing them.

The outcomes investigated in this study were incident cases of CVD and included both deaths and hospital admissions for these causes. ICD-9 classification is used for hospital admission in Italy and ICD-10 is employed for deaths. The following codes were used to identify cases: CVD (ICD-9: 390–459; ICD- 10: I00–I99), excluding hospitalisations for varicose veins (ICD-9: 454, 456) and haemorrhoids (ICD-9: 455), because these are minor disorders already known to be caused or worsened by pregnancy [30–32].

The study population was restricted to subjects 25–45 years, in order to minimise misclassification on the number of men’s and women’s children, because we did not have information about their parity, but rather the presence of children living in the same household. By including only relatively young men and women we diminish the risk that part of the offspring might have already left home. The residual possibility that we have missed out on some children is very small considering that for the cohorts under study at least, the average mother’s age at first birth was 28 years and the average age that children leave home was 26 for women and 29 for men in Italy [33].

Men and women living in households with children derived from previous marriages were excluded (*n* = 216 and *n* = 273, respectively) in order to reduce residual confounding by social circumstances, thereby leaving 20,904 men and 25,246 women available for the analyses.

### Exposure

Parity was assessed by identifying children who were present in the household through a reconstruction of all family members from the NHIS data, including only children from the current marriage (see above). Number of children was examined both as a continuous and a categorical measure (0, 1, 2, 3 + children), while the sex of the offspring was also considered in a further analysis (see Data analysis).

### Covariates

Information about socio-demographics and on exposure to behavioural (physical inactivity, smoking, overweight/obesity) and biological CVD risk factors (diabetes, hypertension) at baseline was drawn from the NHIS.

Maximum educational level in the couple, as an indicator of socioeconomic status, was classified into three categories: low secondary school or less (8 years), high school diploma (13 years), and university degree (≥ 17 years).

Information on geographic area of residence, which is also made available in the NHIS, was categorised in three areas (north, (8 regions), centre (4 regions), south (8 regions).

Physical activity during leisure time (LTPA) practiced regularly was assessed through self-reports asking participants about leisure time activities performed, corresponding to light activity (walking, climbing stairs, etc.), moderate activity (jogging, gym, biking, gardening, etc.), or intense activity (sports: competitive or not). An overall LTPA classification was assigned based on the highest level of activity reported. Further details on the assessment of LTPA in the two cohorts can be found elsewhere [34].

A smoking habit was classified that divided subjects into never, former and current smokers.

Overweight and obesity were assessed by computing the body mass index (BMI) from self-reported height and weight in the survey. According to the WHO classification, BMI was categorised according to: normal weight or underweight (BMI < 25), overweight (25 <  = BMI < 30) and obese (BMI >  = 30) [35].

The Physical Component Summary (PCS), constructed from the SF-12 questionnaires [36], was used as a health indicator at baseline (with a higher score corresponding to better physical health).

Employment status was categorised in four groups: 1) housewives (only women, given that no married men reported to be a housekeeper); 2) employed; 3) unemployed; 4) retired or in other condition.

Number of household members other than husbands, wives and their children, was categorised in three groups (0, 1 and 2 + other members), while presence of members with severe physical limitations was kept as a dichotomous variable, because of the low number of subjects with disabilities in these families (yes/no).

Physical activity during housekeeping, assessed through self-reports, was also considered as a covariate and was divided into two categories (low- or medium/high physical activity in domestic work).

### Data analysis

Missing data on exposures and covariates were imputed by Istat with automatic imputation methods. Details of the imputation process are provided at https://www.istat.it/en/methods-and-tools/methods-and-it-tools/process/processing-tools/concordjava. The proportion of imputed data for the whole surveys was 2.5% in 1999–2000 and 2.8% in 2004–2005.

Descriptive statistics were computed as proportions of subjects in each covariate’s category according to sex and number of children (0, 1, 2, 3 + child); these categories were then compared through chi squared test, while means were compared across the four groups through a one-way analysis of variance for PCS, which was kept as a continuous measure.

Parity was operationalised in different ways in the analyses on the association between parity and CVD risk: 1) ever parity; 2) number of children (0, 1, 2, 3 + children); 3) combinations of number and sex of children in the household (1 son, 1 daughter, 1 son and 1 daughter, >  = 2 sons, >  = 2 daughters, >  = 2 sons and >  = 2 daughters).

Cox proportional hazards models were used to estimate hazard ratios (HR) of incidence of CVD by the different indicators for number of children in the household and for combinations of both number and sex of children.

In a first regression model (Model 1), analyses were adjusted for age, treated as a continuous time-varying variable, for geographic area of residence, year of the baseline survey and maximum educational level within the couple. In a second model (Model 2), further adjustments were made for smoking, physical activity, BMI, diabetes (yes/no), hypertension (yes/no) and for the SF-12 Physical Component Summary, kept as a continuous variable. In a third model (Model 3), household characteristics were also taken into account (i.e., number of other household components, presence of household members with severe physical limitations (yes/no), and whether subjects were engaged in medium/heavy domestic work (yes/no variable)). In the second and the third model, age was also the only covariate treated as a continuous time-varying variable.

Trends in risks across ordered exposure categories of number of children (0, 1, 2, 3 +) were estimated by treating this categorical variable as continuous.

The modifying effect of socioeconomic position on the relationship between parity and CVD risk was examined testing in Model 1 (adjusted for age, area of residence, year of the baseline survey, employment status) the interaction of number of children (in categories: 0, 1, 2, 3 +) both with the maximum educational level within the couple, divided in two categories (high education: high school diploma or university degree; low education: low secondary or elementary school) and with the maximum occupational class within the couple (white or blue collars).

We also investigated effect modification according to employment status, also using Model 1, through an analysis that was restricted to women who were either employed or housewives; this was performed to evaluate consistency with the findings of [26] that the association with number of children was limited to employed women.

After conducting preliminary analyses, which showed only a little influence of covariates on the relationship between parity and CVD, the interactions of parity with education, occupational class and employment status were tested in models with minimal adjustment to preserve statistical power (Model 1: age, area of residence and year of the baseline survey, plus employment status when evaluating the interaction with highest family educational level, and highest educational level in the couple when examining the interaction with employment status).

For all analyses, associations with *p*-values below 0.05 (two-tails) were considered statistically significant.

The analyses were performed using the statistical software Stata, version 18.

## Results

### Descriptive statistics

Frequency distributions of the covariates used in the analyses are presented in Online Appendix Tables A1 and A2 according to categories of children in the household (0, 1, 2, 3 + children) for men and women, respectively.

Men and women with a greater number of children were more likely to be older, in lower SEP (both using highest educational level or occupational class of the couple), living in the South of the country, overweight or obese, less physically active, were more frequently affected by hypertension and diabetes, and showed higher CVD incidence. Age-standardised CVD cumulative incidence of men and women with three or more children was 57% and 73% higher, respectively, when compared to men and women with no children. Most other covariates were also significantly different across categories of number of children in both genders or in only one, because of the large sample, although differences in their frequency distribution were quite small, such as for year of baseline survey, smoking habit, perceived health and presence of other household members.

### Associations between CVD incidence and parity

#### Men

A non-significantly increased risk of CVD was observed for ever parity (HR = 1.11, 95% CI: 0.96–1.32), while a significant association was estimated for number of children examined as a continuous variable (HR = 1.08, 95% CI: 1.02–1.15, for an increase in one child) in models adjusted for age, area of residence, cohort and education (Table 1, Model 1). Only men with three or more children showed a significantly increased risk of CVD (HR = 1.26, 95% CI: 1.02–1.56) when the number of children was examined in categories (0, 1, 2, >  = 3 children), compared to those without children, but there was a significant trend in risk across ordered exposure categories (*p* = 0.02). Considering the children’s sex, only men with 2 or more sons were at significantly higher risk, compared to those without children (HR = 1.24, 95% CI: 1.03–1.51). Restricting the analysis to men with children, an increase of one child was associated with a marginally significant increased CVD risk by 8% (HR = 1.08, 95% CI: 1.00–1.17).

**Table 1:** Hazard Ratios (HR) of cardiovascular diseases (CVD) associated with ever parity, number of children in the household (continuous and categorical), and with combinations of number of children and their sex – Cox regression models. Men 25–45 at baseline

Exposure	n. CVD cases	Model 1		Model 2		Model 3	
		HR	95% CI	HR	95% CI	HR	95% CI
Ever parity	1,226	1.11	0.95–1.30	1.09	0.94–1.28	1.08	0.93–1.26
Number of children^a^	-	**1.08**	**1.02–1.15**	**1.07**	**1.00–1.13**	**1.06**	**1.00–1.13**
0 children (ref.)	196	1		1	-	1	-
1 child	416	1.05	0.88–1.24	1.04	0.88–1.24	1.03	0.87–1.22
2 children	625	1.14	0.97–1.35	1.12	0.95–1.33	1.11	0.94–1.31
3 + children	181	**1.26**	**1.02–1.56**	1.20	0.97–1.49	1.19	0.96–1.48
0 children (ref.)	196	1	-	1	-	1	-
1 son	220	1.07	0.88–1.30	1.06	0.87–1.29	1.05	0.87–1.28
1 daughter	196	1.03	0.84–1.25	1.02	0.84–1.24	1.01	0.82–1.23
1 son and 1 daughter	321	1.16	0.96–1.39	1.13	0.94–1.35	1.11	0.92–1.34
> = 2 sons	279	**1.24**	**1.03–1.51**	**1.21**	**1.00–1.47**	1.20	0.99–1.46
> = 2 daughters	179	1.09	0.89–1.33	1.07	0.87–1.31	1.06	0.86–1.30
> = 2 sons and > = 2 daughters	7	0.87	0.41–1.86	0.89	0.42–1.90	0.87	0.41–1.86

Model 1: adjusted for age, geographic area of residence, educational level, employment status, year of the baseline survey. Model 2: further adjusted for diabetes, hypertension, SF-12 Physical Component Summary, smoking, leisure physical activity, BMI. Model 3: further adjusted for medium/heavy domestic work, number of other household members, presence of components with severe physical limitations. Bold font indicates significance at the 5% level

^a^Continuous variable

Further adjustment for perceived health, education, behavioural and biological risk factors (Table 1, Model 2), as well as for exposure to household factors (Table 1, Model 3), slightly attenuated the risk estimates, with the HR for three children becoming only marginally significant (fully adjusted model: HR = 1.20, 95% CI: 0.97–1.49).

Stratifying the analysis according to dichotomised educational level (Table 2), the HR for having three or more children was higher among the lower educated (HR = 1.29, 95% CI: 0.96–1.75) compared to men with a higher education (HR = 1.11, 95% CI: 0.80–1.53), albeit not significantly (Table 2). Using occupational class as an SEP indicator (Table 3.), the associations increased and became significant among blue collars with two (HR = 1.35, 95% CI: 1.03–1.76) and three or more children (HR = 1.56, 95% CI: 1.14–2.14).

**Table 2:** Hazard Ratios (HR) of cardiovascular diseases associated with number of children in the household, stratified by gender and educational level^1^ – Cox regression models^2^. Analyses restricted to married men and women with or without children. Men and women 25–45 at baseline

**Exposure**	**High education—men**		**Low education—men**			**High education—women**		**Low education—women**		
	**HR**	**95% CI**	**HR**	**95% CI**	***p*-value**^**3**^	**HR**	**95% CI**	**HR**	**95% CI**	***p*-value**^**3**^
Ever parity (ref. no children)	1.14	0.93–1.39	1.12	0.88–1.43	0.92	1.12	0.83–1.51	**1.85**	**1.20–2.86**	**0.03**
0 children (ref.)	1	-	1	-		1	-	1	-	
1 child	1.02	0.81–1.27	1.08	0.83–1.41	0.74	0.99	0.71–1.38	**1.64**	**1.04–2.60**	0.09
2 children	1.19	0.95–1.49	1.06	0.82–1.38	0.40	1.13	0.82–1.57	**2.11**	**1.36–3.29**	**0.02**
3 + children	1.11	0.80–1.53	1.29	0.96–1.75	0.44	1.24	0.81–1.92	**1.88**	**1.16–3.04**	0.15

^1^Education level dichotomised in high (high school, university degree) and low (elementary, low secondary)

^2^Adjusted for age, area of residence, year of the baseline survey, employment status

^3^*p*-value of the interaction test. Bold font indicates significance at the 5% level

**Table 3:** Hazard Ratios (HR) of cardiovascular diseases associated with number of children in the household, stratified by gender and occupational social class^1^ – Cox regression models^2^. Analyses restricted to married men and women with or without children. Men and women 25–45 at baseline

**Exposure**	**High occupational class—men**		**Low occupational class—men**			**High occupational class—women**		**Low occupational class—women**		
	**HR**	**95% CI**	**HR**	**95% CI**	***p*-value**^**2**^	**HR**	**95% CI**	**HR**	**95% CI**	***p*-value**^**2**^
Ever parity (ref. no children)	1.07	0.88–1.31	**1.31**	**1.01–1.68**	0.29	1.15	0.85–1.56	**1.89**	**1.21–2.95**	0.06
0 children (ref.)	1	-	1	-		1	-	1	-	
1 child	1.03	0.83–1.29	1.19	0.90–1.58	0.45	1.04	0.74–1.45	**1.63**	**1.02–2.62**	0.10
2 children	1.09	0.88–1.36	**1.35**	**1.03–1.76**	0.32	1.27	0.92–1.75	**2.00**	**1.26–3.15**	0.10
3 + children	1.21	0.89–1.64	**1.56**	**1.14–2.14**	0.35	1.07	0.69–1.66	**2.21**	**1.35–3.62**	**0.03**

^1^Occupational class dichotomised in high (white collars) and low (blue collars)

^2^Adjusted for age, area of residence, year of the baseline survey, education level

^3^*P*-value of the interaction test. Bold font indicates significance at the 5% level

#### Women

Among women, both ever parity (HR = 1.32, 95% CI: 1.04–1.68) and number of children kept continuous (HR = 1.14, 95% CI: 1.05–1.23, for each child) were positively associated with CVD risk in the analysis controlled for age, area of residence, cohort and education (Table 4., Model 1). Compared to women without children, mothers with one child showed a non-significantly higher risk (HR = 1.18, 95% CI: 0.91–1.54), while those with either two or three or more children both showed a significant increase in CVD risk of around 40%, with a significant trend in risk across ordered exposure categories (*p* < 0.01). Considering the children’s sex, HRs were higher than those of women with no children for all offspring categories, although low and non-significant for having one son, or one daughter, or two sons; in contrast, women with one son and one daughter (HR = 1.53, 95% CI: 1.17–2.00), or two or more daughters (HR = 1.44, 95% CI: 1.08–1.93), or two or more sons and daughters (HR = 2.19, 95%: 1.12–4.27) were at a significantly higher risk. Further adjustment for lifestyles and biological CVD risk factors, and for domestic characteristics, produced little changes in the risk estimates among women (Table 4., Models 2 and 3). Restricting the analysis to women with children, an increase of one child was associated with a significantly increased risk of CVD by 11% (HR = 1.11, 95% CI: 1.01–1.22).

**Table 4:** Hazard Ratios (HR) of cardiovascular diseases (CVD) associated with ever parity, number of children in the household (continuous and categorical), and with combinations of number of children and their sex – Cox regression models. Women 25–45 at baseline

Exposure	n. CVD cases	Model 1		Model 2		Model 3	
		**HR**	**95% CI**	**HR**	**95% CI**	**HR**	**95% CI**
Ever parity	747	**1.32**	**1.04–1.68**	**1.30**	**1.02–1.66**	**1.32**	**1.04–1.69**
Number of children^a^	**-**	**1.14**	**1.05–1.23**	**1.13**	**1.05–1.23**	**1.14**	**1.05–1.23**
0 children (ref.)	75	1	-	1	-	1	
1 child	221	1.18	0.91–1.54	1.17	0.89–1.52	1.18	0.90–1.54
2 children	406	**1.42**	**1.10–1.83**	**1.41**	**1.10–1.82**	**1.44**	**1.12–1.86**
3 + children	120	**1.39**	**1.03–1.87**	**1.35**	**1.00–1.83**	**1.38**	**1.02–1.86**
0 children (ref.)	75	1	-	1	-	1	-
1 son	109	1.11	0.83–1.50	1.09	0.81–1.47	1.11	0.82–1.49
1 daughter	112	1.26	0.94–1.69	1.25	0.93–1.68	1.26	0.94–1.69
1 son and 1 daughter	224	**1.53**	**1.17–2.00**	**1.52**	**1.16–1.99**	**1.55**	**1.18–2.02**
> = 2 sons	148	1.22	0.92–1.63	1.22	0.91–1.62	1.24	0.93–1.65
> = 2 daughters	144	**1.44**	**1.08–1.93**	**1.41**	**1.06–1.89**	**1.44**	**1.08–1.92**
> = 2 sons and > = 2 daughters	10	**2.19**	**1.12–4.27**	**2.15**	**1.10–4.20**	**2.19**	**1.12–4.29**

Model 1: adjusted for age, geographic area of residence, educational level, year of the baseline survey, employment status. Model 2: further adjusted for diabetes, hypertension, SF-12 Physical Component Summary, smoking, leisure physical activity, BMI. Model 3: further adjusted for medium/heavy domestic work, number of other household members, presence of components with severe physical limitations. Bold font indicates significance at the 5% level

^a^Continuous variable

The stratification of Model 1 by educational level revealed striking differences in HRs between women with different education levels. Risks associated with ever parity and number of children in the household were low among those with a higher level of education and were not significantly different from those of women without children (Table 2, 3^rd^ column). By contrast, all HRs were higher and statistically significant (ever parity: HR = 1.89; one child: HR = 1.64; two children: HR = 2.11; three or more children: HR = 1.88) among women with lower education (Table 2, 4th column), with significant or marginally significant interactions of low education with ever parity (*p* = 0.03), and with either one (*p* = 0.09) or two children (*p* = 0.02). Using highest occupational social class in the family as an SEP stratification variable (Table 3.) produced results similar, or even slightly higher, than those obtained using highest educational level.

Stratifying the analyses by employment status (Table 5.) resulted in stronger associations with number of children among housewives (37% of the sample) than among employed women (53% of the sample), in contrast with our expectations, although differences between HRs were not significant (lowest *p*-value for interaction: 0.20).

**Table 5:** Hazard Ratios (HR) of cardiovascular diseases associated with number of children in the household, by employment status – Cox regression models. ^1^ Women 25–45 at baseline

**Exposure**		**Housewives—Women**			**Employed – Women**		
	**n. cases**	**HR**	**95% CI**	**n. cases**	**HR**	**95% CI**	***p*-value**^2^
Ever parity (ref. no children)	**342**	**1.76**	**1.03–3.00**	345	1.18	0.88–1.60	0.20
0 children (ref.)	14	1	-	52	1	-	-
1 child	79	1.60	0.91–2.83	117	1.02	0.73–1.42	0.27
2 children	**197**	**2.02**	**1.17–3.50**	174	1.20	0.87–1.66	0.20
3 + children	**66**	**1.88**	**1.05–3.37**	44	1.25	0.82–1.92	0.49

^1^Adjusted for age, area of residence, year of the baseline survey, and highest educational level in the couple

^2^*P*-value of the interaction test. Bold font indicates significance at the 5% level

## Discussion

The present study found that women with children were at higher risk of developing CVD, no matter whether ever parity or number of children are used, kept continuous or categorical, even though only women with at least two children were at a significantly higher risk for the latter, compared to being nulliparous. Furthermore, limiting the analyses to women with children, each additional child increased CVD incidence by approximately 11%. Therefore, our results support the presence of a positive linear relationship between parity and CVD risk, as reported previously by a few other studies [12, 37–39]. Nevertheless, substantial heterogeneity exists in the literature on such relationship, as some studies reported no or very low associations [40–42] or a higher risk among childless women [43], while others found a J-shaped relation, with the lowest risk among women with 1–2 children, a slightly higher risk among those with 0–1 children and a substantially higher risk for those with more than two children [11, 21, 44, 45].

In spite of such heterogeneity of the results, a linear relationship is also suggested by several studies examining carotid thickness in women, as an indicator of atherosclerosis progression, where strong associations have been observed with parity in different populations, with linear or dose–response relationships with increasing numbers of children [46–53].

Adjustments for behavioural factors (BMI, smoking, physical activity), hypertension and diabetes produced little changes in the associations with number of children, thereby indicating that the higher CVD risks observed among parous women are unlikely to be explained by the mediating role played by these factors; this is consistent with other studies [11, 13, 18, 38].

The relationship with children in the household was less clear among men, even though CVD risk was significantly associated with number of children, kept continuous, and with having three or more children, but not with ever parity. Associations between number of children and CVD risk were in the same direction in both sexes, without significant differences, in accordance with several other studies [11, 18–21], which would indicate an effect of childrearing, rather than childbearing. Support for this interpretation is also provided by the American Nurses’ Health Study, one of the largest prospective investigations into the risk factors for major chronic diseases in women and in which intense child caring was found to be associated, in models adjusted for several potential confounders, with a 50% higher risk of coronary heart disease, not just among women providing care to their children, but also among grandmothers who provided care to their grandchildren [54]. Furthermore, in a US study, the most common pregnancy complications (low birth weight, preterm delivery, gestational hypertension and preeclampsia) were found to mediate only about 10% of the relationship between parity and CVD prevalence among older women [12]. Conversely, it is worth noting that one study observed a linear positive association between number of children and CVD mortality in women, but not in men [23], while two studies found stronger associations between carotid intima-media thickness and parity among women than among men [50, 51]; these findings also suggest that factors linked to pregnancy play a role.

Stratification of the analyses by socioeconomic position using highest educational level within the couple, as the stratification variable, indicated stronger associations with number of children among both women and men with lower SEP, compared to those with higher SEP, with a significant interaction among women between low education and both ever parity and having two children. Our results are in line with those of [17], who found an association between higher parity and CVD mortality that was limited to women with low education.

Similar or even stronger differences in terms of SEP were observed when occupational social class was used as the social stratification variable, particularly among men, consistently with the observation that in several European cohorts it was a better predictor of mortality than educational level [55], being an indicator closer in time, which would represent the individuals’ actual socioeconomic position better. The consistency of HRs, using either education or occupational class, suggests a sufficient level of validity in these socioeconomic indicators and of the SEP gradient obtained in the stratified analysis.

The stronger associations found in women with low SEP between number of children and CVD also supports the hypothesis that the excess risk is more likely to be attributable to factors linked to childrearing, the effects of which would be moderated by the availability of higher education, income or wealth in women belonging generally to more socially advantaged families. Such differences in risk by SEP could be explained by the greater mental and physical burden to which low SEP women may be exposed in raising children, possibly because of lower educational resources with which to manage conflicts with children, as well as the lower possibility of buying private services for the household and for their children, such as babysitting, housekeeping, private full-time schools, sport and social activities and household amenities; the presence of such resources may buffer women’s physical and mental workload related to childcare and domestic chores.

A possible concern is that the higher risk of CVD associated with parity and number of children observed among lower SEP women could, in theory, be attributable to a higher prevalence of pregnancy complications in this group. However, previous studies investigating the existence of a socioeconomic gradient for pregnancy complications have produced mixed results for the most common types of pregnancy complications, including preterm birth [56–59], preeclampsia or eclampsia [60–62], gestational hypertension [58, 60, 62, 63] and gestational diabetes [64–66]. They do not seem able to explain the observed differences in CVD risks associated with higher parity between low- and high-educated women in our study, even though the CVD risks associated with these pregnancy outcomes are mainly around two-fold and that their overall prevalence among parous women would not be greater than 20–30% [8].

In contrast, no effect modification by employment status was observed among women, unlike the results of a couple of studies on coronary heart disease in which the association with number of children was limited to the employed [25, 26]. Furthermore, HRs were even higher among among housewives than among employed women, even though among housewives with no children the number of CVD events was quite low (*n* = 14), so that risk estimates were affected by substantial uncertainty. Therefore, our results do not support the “double burden” hypothesis of an interaction between paid employment and number of children on CVD; this is consistent with the findings from the Nurses’ Health Study [54], the only other study to our knowledge, together with the two others cited above, that have evaluated effect modification in terms of employment.

The associations between CVD and number of children were not significantly different by gender, but HRs among women were almost the double those found among men, thereby suggesting that childbearing may be responsible for at least part of the excess risk of CVD in women with children. However, other household factors associated with childcare could also explain such a difference, in particular the time spent in domestic work and childrearing, which appears to be disproportionately higher among women than among men in Italy. For example, a study conducted by the Organization for Economic Cooperation and Development (OECD) reported that women in Italy perform 11 h more domestic work than men per week [67], while in a national survey on time use, domestic work was found to be entirely accomplished by Italian women in 41% of the couples examined [68].

### Strengths

This study’s main strengths include the large cohorts used, which were representative at the national level and allowed us to generalise our results to the Italian population of corresponding age, as well as the detailed information available at baseline on household characteristics and on many potential confounders, including several behavioural and biological CVD risk factors, perceived health, different measures of socioeconomic position, employment status, number of family components and their physical limitations; all of this allowed us to control for all of these covariates in the analyses conducted.

### Limitations

The study’s main limitation is that the assessment of the number of children was done through codes identifying each type of family member (e.g., father, mother, children, grandparents, others) living in the household at the time of the interview. Therefore, we could have missed children who had already left home at the time of the surveys. However, we expect that the proportion of missing children was rather low, given that the study population was restricted to parents up to 45 years, considering that in Italy in the generations born in the 60 s-80 s (like those in the study population) mean age at first child was high (above 30 years for men and 28 years for women), that Italian children are characterised by an elevated age at which they leave home (median age: 29 years for men and 26 years for women) and that almost 90% of children had not left home for the first time before turning 20 years old [33].

In spite of the large cohorts used, the restriction to a younger population limited the number of CVD events observed during follow-up, with a corresponding, reduced statistical power of the analyses, which prevented us from examining the association between parity and specific CVD causes, such as coronary heart disease, stroke or heart failure, as is done in other studies. CVDs are a quite heterogeneous group, even though many cardiovascular diseases share a common pathogenesis (i.e., the atherosclerotic process), which includes many pathologies that are likely characterised by other underlying mechanisms, such as arrhythmias for example. Among these, it is worth noting that atrial fibrillation has also been associated with higher parity in a couple of studies [69, 70]. Therefore, our results must be interpreted with caution, although the exclusion from the outcome of hospitalisations for varicose veins and haemorrhoids is expected to have allowed for the analyses to focus on more relevant cardiovascular diseases, given that these are minor CVD events which, nonetheless, constituted a substantial proportion of total CVD hospitalisations in this relatively young sample.

Another limitation concerns the possible misclassification of the number of children of the women enrolled, deriving from the impossibility of reliably detecting newborns during follow-up (and, therefore, also the change in the condition of the mother). Therefore, the measurement of number of children was surely subject to error, with the expected consequence of a non-differential misclassification of parity leading to an underestimate of the associated hazards ratios (attenuation or dilution bias) [71].

## Conclusions

In conclusion, the present study found an association between higher parity and CVD risk in both genders, as well as a higher risk of CVD associated with higher parity in men and women of lower SEP, both indicating that childrearing would play an important role in CVD development. However, the stronger associations with parity observed among women than men, especially those in low SEP, suggest that part of the association with parity may be attributable to the effect of childbearing.

Nevertheless, the findings do not support the “double burden” hypothesis, thereby proving that it is not the combination of paid work and family responsibilities per se that overloads mothers physically and mentally, but rather insufficient material and immaterial resources, conveyed by lower education and lower occupational class, which limit a person’s actual ability to cope with the physical and psychological stressors associated with childrearing.

Our results indicate the need for larger welfare system interventions in support of parents, in particular among those in more disadvantaged social positions, in the area of childcare services and economic transfers in specific.

Future research should aim to clarify the respective roles played by childrearing and childbearing in the development of maternal CVD and should try to shed light on pregnancy complications’ potential confounding effect.

### Supplementary Information

**Additional file 1: Online Appendix: Table A1.** Characteristics of married men aged 25 to 45 years included in the study, by children categories (0, 1, 2, 3+ children). **Table A2.** Characteristics of married women aged 25 to 45 years included in the study, by children categories (0, 1, 2, 3+ children). 

## Data Availability

Data used for the analysis are subjected to the legal restrictions established by the European privacy law, as the data contain potentially identifying or sensitive patient information. Open access to data is not possible but collaborations in specific projects with other research groups or institutes are possible upon collaboration agreement approval from the Presidential Committee of the ISTAT. Further requests of information on the project and on collaborations can be addressed to the authors and/or to the ISTAT officer responsible for the data used in this study, Dr. Gabriella Sebastiani (gabriella.sebastiani@istat.it).
