# Supplementary material for: Child rearing or childbearing? Risk of cardiovascular diseases associated to parity and number of children
Source: BMC Public Health. 2024 Jan 23;24:272. doi: 10.1186/s12889-023-17119-z (PMC10804732; doi:10.1186/s12889-023-17119-z)
Supplement: Supplementary file 1 — Additional file 1: Online Appendix: Table A1. Characteristics of married men aged 25 to 45 years included in the study, by children categories (0, 1, 2, 3+ children). Table A2. Characteristics of married women aged 25 to 45 years included in the study, by children categories (0, 1, 2, 3+ children). [file 12889_2023_17119_MOESM1_ESM.docx]

# Online Appendix

**Table A1.** Characteristics of married men aged 25 to 45 years included in the study, by children categories (0, 1, 2, 3+ children)

|  | **No children** | | | **1 child** | | | **2 children** | | | **3+ children** | | | **Total** | | | **p-value** |
| --- | --- | --- | --- | --- | --- | --- | --- | --- | --- | --- | --- | --- | --- | --- | --- | --- |
|  | | **N** | **%** | | **N** | **%** | | **N** | **%** | | **N** | **%** | | **N** | **%** |  |
| **Cohort** | |  |  | |  |  | |  |  | |  |  | |  |  |  |
| 2000 | | 2,188 | 54.0 | | 3,868 | 56.0 | | 4,197 | 52.0 | | 938 | 49.9 | | 11,191 | 53.5 | <0.001 |
| 2005 | | 1,863 | 46.0 | | 3,038 | 44.0 | | 3,871 | 48.0 | | 941 | 50.1 | | 9,713 | 46.5 |  |
| **Age** | |  |  | |  |  | |  |  | |  |  | |  |  |  |
| 25-29 | | 658 | 16.2 | | 510 | 7.4 | | 159 | 2.0 | | 22 | 1.2 | | 1,349 | 6.5 | <0.001 |
| 30-34 | | 1,439 | 35.5 | | 1,768 | 25.6 | | 1,009 | 12.5 | | 142 | 7.6 | | 4,358 | 20.9 |  |
| 35-39 | | 1,087 | 26.8 | | 2,278 | 33.0 | | 2,694 | 33.4 | | 569 | 30.3 | | 6,628 | 31.7 |  |
| 40-45 | | 867 | 21.4 | | 2,350 | 34.0 | | 4,206 | 52.1 | | 1,146 | 61.0 | | 8,569 | 41.00 |  |
| **Highest educational level of the couple** | |  |  | |  |  | |  |  | |  |  | |  |  |  |
| University degree | | 769 | 19.0 | | 983 | 14.2 | | 998 | 12.4 | | 179 | 9.5 | | 2,929 | 14.0 | <0.001 |
| High school diploma | | 1,801 | 44.5 | | 2,989 | 43.3 | | 3,133 | 38.8 | | 544 | 29.0 | | 8,467 | 40.5 |  |
| Low secondary or less | | 1,481 | 36.6 | | 2,934 | 42.5 | | 3,937 | 48.8 | | 1,156 | 61.5 | | 9,508 | 45.5 |  |
| **Highest occupational class of the couple** | |  |  | |  |  | |  |  | |  |  | |  |  |  |
| Employers, professionals, executives,  office workers | | 2,323 | 57.3 | | 3,731 | 54.0 | | 4,064 | 50.4 | | 741 | 39.4 | | 10,859 | 52.0 | <0.001 |
| Blue collars and self-employed | | 1,669 | 41.2 | | 3,089 | 44.7 | | 3,907 | 48.4 | | 1,101 | 58.6 | | 9,766 | 46.7 |  |
| missing | | 59 | 1.5 | | 86 | 1.3 | | 97 | 1.2 | | 37 | 2.0 | | 279 | 1.3 |  |
| **Geographical area** | |  |  | |  |  | |  |  | |  |  | |  |  |  |
| North | | 2,107 | 52.0 | | 3,001 | 43.5 | | 2,827 | 35.0 | | 471 | 25.1 | | 8,406 | 40.2 | <0.001 |
| Center | | 700 | 17.3 | | 1,305 | 18.9 | | 1,257 | 15.6 | | 192 | 10.2 | | 3,454 | 16.5 |  |
| South | | 1,244 | 30.7 | | 2,600 | 37.7 | | 3,984 | 49.4 | | 1,216 | 64.7 | | 9,044 | 43.3 |  |
| **Smoking** | |  |  | |  |  | |  |  | |  |  | |  |  |  |
| Never smoker | | 1,721 | 42.3 | | 2,771 | 40.1 | | 3,131 | 38.8 | | 694 | 36.9 | | 8,317 | 39.8 | <0.001 |
| Former smoker | | 817 | 20.2 | | 1,592 | 23.1 | | 1,952 | 24.2 | | 439 | 23.4 | | 4,800 | 23.0 |  |
| Current smoker | | 1,513 | 37.4 | | 2,543 | 36.8 | | 2,985 | 37.0 | | 746 | 39.7 | | 7,787 | 37.3 |  |
| **Body Mass Index** | |  |  | |  |  | |  |  | |  |  | |  |  |  |
| Normal or underweight | | 2,102 | 51.9 | | 3,259 | 47.2 | | 3,422 | 42.4 | | 694 | 36.9 | | 9,477 | 45.3 | <0.001 |
| Overweight | | 1,611 | 39.8 | | 3,008 | 43.6 | | 3,846 | 47.7 | | 943 | 50.2 | | 9,408 | 45.0 |  |
| Obese | | 338 | 8.3 | | 639 | 9.3 | | 800 | 9.9 | | 242 | 12.9 | | 2,019 | 9.7 |  |
| **Hypertension - yes** | | 165 | 4.1 | | 342 | 5.0 | | 480 | 6.0 | | 108 | 5.8 | | 1,095 | 5.2 | <0.001 |
| **Diabetes - yes** | | 29 | 0.7 | | 62 | 0.9 | | 90 | 1.1 | | 25 | 1.3 | | 206 | 1.0 | 0.06 |
| **Physical activity status** | |  |  | |  |  | |  |  | |  |  | |  |  |  |
| Intense | | 892 | 22.0 | | 1,130 | 16.4 | | 1,081 | 13.4 | | 181 | 9.6 | | 3,284 | 15.7 | <0.001 |
| Regular | | 1,092 | 27.0 | | 1,772 | 25.7 | | 1,843 | 22.8 | | 345 | 18.4 | | 5,052 | 24.2 |  |
| Light | | 856 | 21.1 | | 1,597 | 23.1 | | 1,845 | 22.9 | | 412 | 21.9 | | 4,710 | 22.5 |  |
| Never | | 1,211 | 29.9 | | 2,407 | 34.9 | | 3,299 | 40.9 | | 941 | 50.1 | | 7,858 | 37.6 |  |
| **Employment status** | |  |  | |  |  | |  |  | |  |  | |  |  |  |
| Employed | | 3,868 | 95.5 | | 6,561 | 95.0 | | 7,655 | 94.9 | | 1,732 | 92.2 | | 19,816 | 94.8 | <0.001 |
| Unemployed | | 143 | 3.5 | | 269 | 3.9 | | 334 | 4.1 | | 121 | 6.4 | | 867 | 4.2 |  |
| Others | | 40 | 1.0 | | 76 | 1.1 | | 79 | 1.0 | | 26 | 1.4 | | 221 | 1.1 |  |
| **Presence of household members with severe** | |  |  | |  |  | |  |  | |  |  | |  |  |  |
| **physical limitations - yes** | | 51 | 1.3 | | 135 | 2.0 | | 255 | 3.2 | | 96 | 5.1 | | 536 | 2.6 | <0.001 |
| **Other household members (except children)** | |  |  | |  |  | |  |  | |  |  | |  |  |  |
| 0 | | 3,916 | 96.7 | | 6,724 | 97.4 | | 7,863 | 97.5 | | 1,821 | 96.9 | | 20,324 | 97.2 | <0.001 |
| 1 | | 75 | 1.9 | | 147 | 2.1 | | 162 | 2.0 | | 52 | 2.8 | | 436 | 2.1 |  |
| 2+ | | 60 | 1.5 | | 35 | 0.5 | | 43 | 0.5 | | 6 | 0.3 | | 144 | 0.7 |  |
|  | |  |  | |  |  | |  |  | |  |  | |  |  |  |
| **Mid-heavy physical domestic work - yes** | | 1,338 | 33.0 | | 1,989 | 28.8 | | 2,078 | 25.8 | | 427 | 22.7 | | 5,832 | 22.7 | <0.001 |
|  | |  |  | |  |  | |  |  | |  |  | |  |  |  |
|  | |  |  | |  |  | |  |  | |  |  | |  |  |  |
|  | | **Mean** | **St. dev.** | | **Mean** | **St. dev.** | | **Mean** | **St. dev.** | | **Mean** | **St. dev.** | | **Mean** | **St. dev.** |  |
| **Physical Component Summary** | | 54.0 | 5.5 | | 53.7 | 5.8 | | 53.4 | 6.0 | | 53.0 | 6.5 | |  |  | <0.001 |
|  | | **Cumul.**  **Incid.** | **95% CI** | | **Cumul.**  **Incid.** | **95% CI** | | **Cumul.**  **Incid.** | **95% CI** | | **Cumul.**  **Incid.** | **95% CI** | | **Cumul.**  **Incid.** | **95% CI** |  |
| **CVD incidence (standardized for age)** | | 6.1 | 5.3-7.0 | | 6.3 | 5.8-7.0 | | 7.2 | 6.7-7.9 | | 9.6 | 7.9-11.6 | | 6.9 | 6.6-7.3 | <0.001 |

**Table A2.** Characteristics of married women aged 25 to 45 years included in the study, by children categories (0, 1, 2, 3+ children)

|  | **No children** | | | | **1 child** | | | | **2 children** | | | | **3+ children** | | | | **Total** | | | | **p-value** | |  |
| --- | --- | --- | --- | --- | --- | --- | --- | --- | --- | --- | --- | --- | --- | --- | --- | --- | --- | --- | --- | --- | --- | --- | --- |
|  | **N** | | **%** | | **N** | | **%** | | **N** | | **%** | | **N** | | **%** | | **N** | | **%** | |  | |  |
| **Cohort** |  | |  | |  | |  | |  | |  | |  | |  | |  | |  | |  | |  |
| 2000 | 2,068 | | 54.8 | | 4,596 | | 56.7 | | 5,540 | | 52.2 | | 1,388 | | 50.4 | | 13,592 | | 53.8 | | <0.001 | |  |
| 2005 | 1,707 | | 45.2 | | 3,515 | | 43.3 | | 5,068 | | 47.8 | | 1,364 | | 49.6 | | 11,654 | | 46.2 | |  |  |  |
| **Age** |  | |  | |  | |  | |  | |  | |  | |  | |  | |  | |  | |  |
| 25-29 | 1,043 | | 27.6 | | 1,206 | | 14.9 | | 559 | | 5.3 | | 83 | | 3.0 | | 2,891 | | 11.5 | | <0.001 | |  |
| 30-34 | 1,203 | | 31.9 | | 2,262 | | 27.9 | | 2,088 | | 19.7 | | 380 | | 13.8 | | 5,933 | | 23.5 | |  |  |  |
| 35-39 | 832 | | 22.0 | | 2,106 | | 26.0 | | 3,582 | | 33.8 | | 932 | | 33.9 | | 7,452 | | 29.5 | |  |  |  |
| 40-45 | 697 | | 18.5 | | 2,537 | | 31.3 | | 4,379 | | 41.3 | | 1,357 | | 49.3 | | 8,970 | | 35.5 | |  |  |  |
| **Highest educational level of the couple** |  | |  | |  | |  | |  | |  | |  | |  | |  | |  | |  | |  |
| University degree | 800 | | 21.2 | | 1,204 | | 14.8 | | 1,313 | | 12.4 | | 261 | | 5.5 | | 3,578 | | 14.2 | | <0.001 | |  |
| High school diploma | 1,668 | | 44.2 | | 3,384 | | 41.7 | | 4,018 | | 37.9 | | 781 | | 28.4 | | 9,851 | | 39.0 | |  |  |  |
| Low secondary or less | 1,307 | | 34.6 | | 3,523 | | 43.4 | | 5,277 | | 49.8 | | 1,710 | | 62.1 | | 11,817 | | 46.8 | |  |  |  |
| **Highest occupational class of the couple** |  | |  | |  | |  | |  | |  | |  | |  | |  | |  | |  | |  |
| Employers, professionals, executives,  office workers | 2,305 | | 61.1 | | 4,502 | | 55.5 | | 5,402 | | 50.9 | | 1,101 | | 40.0 | | 13,310 | | 52.7 | | <0.001 | |  |
| Blue collars and self-employed | 1,387 | | 36.7 | | 3,453 | | 42.6 | | 5,005 | | 47.2 | | 1,571 | | 57.1 | | 11,416 | | 45.2 | |  |  |  |
| missing | 83 | | 2.2 | | 156 | | 1.9 | | 201 | | 1.9 | | 80 | | 2.9 | | 520 | | 2.1 | |  |  |  |
| **Geographical area** |  | |  | |  | |  | |  | |  | |  | |  | |  | |  | |  | |  |
| North | 1,960 | | 51.9 | | 3,625 | | 44.7 | | 3,661 | | 34.5 | | 677 | | 24.6 | | 9,923 | | 39.3 | | <0.001 | |  |
| Center | 639 | | 16.9 | | 1,528 | | 18.8 | | 1,702 | | 16.0 | | 266 | | 9.7 | | 4,135 | | 16.4 | |  |  |  |
| South | 1,176 | | 31.2 | | 2,958 | | 36.5 | | 5,245 | | 49.4 | | 1,809 | | 65.7 | | 11,188 | | 44.3 | |  |  |  |
| **Smoking** |  | |  | |  | |  | |  | |  | |  | |  | |  | |  | |  | |  |
| Never smoker | 2,391 | | 63.3 | | 4,921 | | 60.7 | | 6,613 | | 62.3 | | 1,761 | | 64.0 | | 15,686 | | 62.1 | | 0.02 | |  |
| Former smoker | 601 | | 15.9 | | 1,410 | | 17.4 | | 1,756 | | 16.6 | | 422 | | 15.3 | | 4,189 | | 16.6 | |  |  |  |
| Current smoker | 783 | | 20.7 | | 1,780 | | 22.0 | | 2,239 | | 21.1 | | 569 | | 20.7 | | 5,371 | | 21.3 | |  |  |  |
| **Body Mass Index** |  | |  | |  | |  | |  | |  | |  | |  | |  | |  | |  | |  |
| Normal or underweight | 3,092 | | 81.9 | | 6,348 | | 78.3 | | 7,771 | | 73.3 | | 1,744 | | 63.4 | | 18,955 | | 75.1 | | <0.001 | |  |
| Overweight | 515 | | 13.6 | | 1,360 | | 16.8 | | 2,216 | | 20.9 | | 775 | | 28.2 | | 4,866 | | 19.3 | |  |  |  |
| Obese | 168 | | 4.5 | | 403 | | 5.0 | | 621 | | 5.9 | | 233 | | 8.5 | | 1,425 | | 5.6 | |  |  |  |
| **Hypertension** - yes | 107 | | 2.8 | | 364 | | 4.5 | | 466 | | 4.4 | | 126 | | 4.6 | | 1,063 | | 4.2 | | <0.001 | |  |
| **Diabetes** – yes | 24 | | 0.6 | | 88 | | 1.1 | | 78 | | 0.7 | | 31 | | 1.1 | | 221 | | 0.9 | | 0.01 | |  |
| **Physical activity status** |  | |  | |  | |  | |  | |  | |  | |  | |  | |  | |  | |  |
| Intense | 543 | | 14.4 | | 635 | | 7.8 | | 684 | | 6.5 | | 104 | | 3.8 | | 1,966 | | 7.8 | | <0.001 | |  |
| Regular | 976 | | 25.9 | | 1,718 | | 21.2 | | 1,955 | | 18.4 | | 385 | | 14.0 | | 5,034 | | 19.9 | |  |  |  |
| Light | 1,091 | | 28.9 | | 2,572 | | 31.7 | | 3,182 | | 30.0 | | 795 | | 28.9 | | 7,640 | | 30.3 | |  |  |  |
| Never | 1,165 | | 30.9 | | 3,186 | | 39.3 | | 4,787 | | 45.1 | | 1,468 | | 53.3 | | 10,606 | | 42.0 | |  |  |  |
| **Employment status** |  | |  | |  | |  | |  | |  | |  | |  | |  | |  | |  | |  |
| Housewives | 707 | | 18.7 | | 2,554 | | 31.5 | | 4,542 | | 42.8 | | 1,477 | | 53.7 | | 9,280 | | 36.8 | | <0.001 | |  |
| Employed | 2,641 | | 70.0 | | 4,662 | | 57.5 | | 5,061 | | 47.7 | | 989 | | 35.9 | | 13,353 | | 52.9 | |  |  |  |
| Unemployed | 335 | | 8.9 | | 768 | | 9.5 | | 850 | | 8.0 | | 223 | | 8.1 | | 2,176 | | 8.6 | |  |  |  |
| Other | 92 | | 2.4 | | 127 | | 1.6 | | 155 | | 1.5 | | 63 | | 2.3 | | 437 | | 1.7 | |  |  |  |
| **Presence of household members with severe** |  | |  | |  | |  | |  | |  | |  | |  | |  | |  | |  | |  |
| **physical limitations - yes** | 50 | | 1.3 | | 191 | | 2.4 | | 374 | | 3.5 | | 159 | | 5.8 | | 774 | | 3.1 | | <0.001 | |  |
| **Other household members (except children)** |  | |  | |  | |  | |  | |  | |  | |  | |  | |  | |  | |  |
| 0 | 3,656 | | 96.9 | | 7,856 | | 96.9 | | 10,285 | | 97.0 | | 2,664 | | 96.8 | | 24,461 | | 96.9 | | <0.001 | |  |
| 1 | 73 | | 1.9 | | 217 | | 2.7 | | 259 | | 2.4 | | 77 | | 2.8 | | 626 | | 2.5 | |  |  |  |
| 2+ | 46 | | 1.2 | | 38 | | 0.5 | | 64 | | 0.6 | | 11 | | 0.4 | | 626 | | 2.5 | |  |  |  |
| **Mid-heavy physical domestic work - yes** | 3,319 | | 87.9 | | 7,519 | | 92.7 | | 10,025 | | 94.5 | | 2,638 | | 95.9 | | 1,745 | | 93.1 | | <0.001 | |  |
|  | **Mean** | | **St. dev.** | | **Mean** | | **St. dev.** | | **Mean** | | **St. dev.** | | **Mean** | | **St. dev.** | | **Mean** | | **St. dev.** | |  | |  |
| **Physical Component Summary** | 52.7 | | 6.9 | | 52.8 | | 6.7 | | 52.9 | | 6.5 | | 52.7 | | 6.7 | | 52.8 | | 6.6 | | 0.23 | |  |
|  | | **Cumul.**  **Incid.** | | **95% CI** | | **Cumul.**  **Incid.** | | **95% CI** | | **Cumul.**  **Incid.** | | **95% CI** | | **Cumul.**  **Incid.** | | **95% CI** | | **Cumul.**  **Incid.** | | **95% CI** | |  | |
| **CVD incidence (standardized for age)** | | 2.6 | | 2.0-3.3 | | 3.2 | | 2.8-3.6 | | 3.9 | | 3.5-4.2 | | 4.5 | | 3.7-5.3 | | 3.5 | | 3.3-3.8 | | <0.001 | |
